# Improving outcome reporting in clinical trial reports and protocols: study protocol for the Instrument for reporting Planned Endpoints in Clinical Trials (InsPECT)

**DOI:** 10.1186/s13063-019-3248-0

**Published:** 2019-03-06

**Authors:** Nancy J. Butcher, Andrea Monsour, Emma J. Mew, Peter Szatmari, Agostino Pierro, Lauren E. Kelly, Mufiza Farid-Kapadia, Alyssandra Chee-a-tow, Leena Saeed, Suneeta Monga, Wendy Ungar, Caroline B. Terwee, Sunita Vohra, Dean Fergusson, Lisa M. Askie, Paula R. Williamson, An-Wen Chan, David Moher, Martin Offringa

**Affiliations:** 1 Child Health Evaluative Sciences, The Hospital for Sick Children Research Institute, Toronto, Canada; 2 Department of Psychiatry, The Hospital for Sick Children, Toronto, Canada; 3 Centre for Addiction and Mental Health, Toronto, Canada; 4 Department of Psychiatry, University of Toronto, Toronto, Canada; 5 Division of General and Thoracic Surgery, The Hospital for Sick Children, Toronto, Canada; 6 Department of Pediatrics and Child Health, Clinical Trials Platform, George and Fay Yee Centre for Healthcare Innovation, University of Manitoba, Winnipeg, Canada; 7 Institute for Health Policy, Management and Evaluation, University of Toronto, Toronto, Canada; 8 Amsterdam UMC, Vrije Universiteit Amsterdam, Department of Epidemiology and Biostatistics, Amsterdam Public Health Research Institute, Amsterdam, Netherlands; 9 The Departments of Pediatrics, Medicine, and Psychiatry, Faculty of Medicine & Dentistry, University of Alberta, Edmonton, Canada; 10 Clinical Epidemiology Program, Ottawa Hospital Research Institute, Ottawa, Canada; 11 Department of Medicine, University of Ottawa, Ottawa, Canada; 12 NHMRC Clinical Trials Centre, University of Sydney, Sydney, Australia; 13 MRC North West Hub for Trials Methodology Research, University of Liverpool, Liverpool, UK; 14 Department of Medicine, Women’s College Research Institute, University of Toronto, Toronto, Canada; 15 Centre for Journalology, Clinical Epidemiology Program, Ottawa Hospital Research Institute, Ottawa, Canada

**Keywords:** Trial, Trial protocol, Reporting guideline, Outcome, Endpoint, SPIRIT, CONSORT

## Abstract

**Background:**

Inadequate and poor quality outcome reporting in clinical trials is a well-documented problem that impedes the ability of researchers to evaluate, replicate, synthesize, and build upon study findings and impacts evidence-based decision-making by patients, clinicians, and policy-makers. To facilitate harmonized and transparent reporting of outcomes in trial protocols and published reports, the Instrument for reporting Planned Endpoints in Clinical Trials (InsPECT) is being developed. The final product will provide unique InsPECT extensions to the SPIRIT (Standard Protocol Items: Recommendations for Interventional Trials) and CONSORT (Consolidated Standards of Reporting Trials) reporting guidelines.

**Methods:**

The InsPECT SPIRIT and CONSORT extensions will be developed in accordance with the methodological framework created by the EQUATOR (Enhancing the Quality and Transparency of Health Research Quality) Network for reporting guideline development. Development will consist of (1) the creation of an initial list of candidate outcome reporting items synthesized from expert consultations and a scoping review of existing guidance for reporting outcomes in trial protocols and reports; (2) a three-round international Delphi study to identify additional candidate items and assess candidate item importance on a 9-point Likert scale, completed by stakeholders such as trial report and protocol authors, systematic review authors, biostatisticians and epidemiologists, reporting guideline developers, clinicians, journal editors, and research ethics board representatives; and (3) an in-person expert consensus meeting to finalize the set of essential outcome reporting items for trial protocols and reports, respectively. The consensus meeting discussions will be independently facilitated and informed by the empirical evidence identified in the primary literature and through the opinions (aggregate rankings and comments) collected via the Delphi study. An integrated knowledge translation approach will be used throughout InsPECT development to facilitate implementation and dissemination, in addition to standard post-development activities.

**Discussion:**

InsPECT will provide evidence-informed and consensus-based standards focused on outcome reporting in clinical trials that can be applied across diverse disease areas, study populations, and outcomes. InsPECT will support the standardization of trial outcome reporting, which will maximize trial usability, reduce bias, foster trial replication, improve trial design and execution, and ultimately reduce research waste and help improve patient outcomes.

**Electronic supplementary material:**

The online version of this article (10.1186/s13063-019-3248-0) contains supplementary material, which is available to authorized users.

## Background

Clinical trials, when appropriately designed, conducted, and reported, are the gold standard study design for generating evidence about treatment efficacy, safety, effectiveness, and efficiency. To be able to critically evaluate and use the results of a trial, however, readers require complete, clear, and transparent information with respect to what was planned, what was done, and what was found [1]. Inadequate reporting of clinical trials is well documented in the medical literature, even with respect to basic methodological details such as the definition of the primary outcome, specification of who was blinded, and explanation of how trial sample size was calculated [2–4]. Such incomplete reporting contributes to significant and avoidable waste of health research investment and impedes reproducibility [5, 6].

In an effort to improve trial reporting quality, reporting guidelines have been developed to standardize the reporting of clinical trial reports and their corresponding protocols. These guidelines include Consolidated Standards of Reporting Trials (CONSORT) [7], first developed in 1996 and updated in 2010, for trial reports published in academic journals, and Standard Protocol Items: Recommendations for Interventional Trials (SPIRIT), developed in 2013, for trial protocols [8]. Numerous extensions have since been developed for CONSORT and SPIRIT, refining their applications to specific populations, study designs, interventions, and contexts [9]. There is evidence that completeness of trial reporting has improved since the development of CONSORT, as shown by increased reporting of CONSORT checklist items, particularly in journals that have endorsed the guideline [10, 11]. CONSORT is now endorsed by more than half of core medical journals, including *The Lancet*, *BMJ*, *JAMA*, and the *New England Journal of Medicine* as well as field-specific journals [12]. However, endorsement policies are not always clear; thus increased effort by journals with respect to enforcement of completion and evaluation of guideline adherence may help further improve the current state of suboptimal trial reporting [10].

Despite the availability and implementation of these well-established reporting guidelines for trials, significant concerns regarding the quality of the reporting of trial outcomes remain [13–18]. In the context of a clinical trial, an outcome refers to what is being measured on trial participants to examine the effect of exposure to a health intervention [19]. SPIRIT and CONSORT provide some general guidance on how to report outcomes [7, 8], including pre-defined primary and secondary outcomes, method of outcome assessment, and timing of outcome assessment. However, outcome reporting remains insufficient across disciplines and academic journals; key information about the selection process, definition, measurement, and analysis of primary outcomes is often missing or poorly reported [2, 3, 13, 20–24]. It has been estimated that up to 60% of trials change, introduce, or omit a primary outcome between protocol and publication of the trial report [20, 25–27], when a protocol is even available for comparison. Less is known about secondary outcomes, which may be even more prone to bias and inadequate reporting [17]. As clinical trials are “only as credible as their outcomes” [28], this lack of transparency and completeness reduces or prevents reproducibility, critical appraisal, knowledge synthesis, and uptake of trial results into clinical practice. Moreover, it enables the introduction of bias into the medical literature by facilitating selective reporting and outcome switching.

Although calls for improved reporting of trial outcomes have been made [13, 14, 16, 29], to date it is unknown what actually constitutes useful, complete reporting of trial outcomes to knowledge users. No evidence- and/or consensus-based detailed guidance currently exists for authors to follow to ensure that their reporting is complete, transparent, and replicable. SPIRIT requires more information on trial outcomes to be reported than CONSORT, but neither reflects, for example, the increasingly widespread attempts to incorporate the patient voice into clinical research. There is no requirement to report why an outcome was selected, to provide a rationale for the way the outcome was defined, or to describe the acceptability to patients of measuring the chosen outcome. The advent of SPIRIT and CONSORT extensions for patient-reported outcomes (PROs) [30, 31] as well as a CONSORT extension for harms [32] represent important steps in improving the reporting of trial outcomes. Recently published guidelines for the content of statistical analysis plans (SAPs) are also now available [33]. However, more comprehensive and generic guidance that is applicable to all outcome types, disease areas, and populations is still needed for trial protocols and published reports.

This protocol outlines the development process for an internationally harmonized outcome reporting standard for clinical trial protocols and reports, called the Instrument for reporting Planned Endpoints in Clinical Trials (InsPECT). Through an evidence-based and consensus-based process, InsPECT will evaluate what constitutes complete reporting of trial outcomes, with respect to outcome selection, rationale, definition, measurement, outcome analysis and its presentation, interpretation, and transparent reporting of any outcome modifications between trial report and protocol. InsPECT will ultimately form two evidence-based reporting extensions, one specific to trial protocols (SPIRIT extension) and one specific to trial reports (CONSORT extension). The InsPECT extensions will be complementary to the work of the core outcome set developers and the Core Outcome Measures in Effectiveness Trials (COMET) Initiative, which provides information and guidance on which outcomes to measure and report for particular health conditions [34].

## Methods

**Fig. 1:**
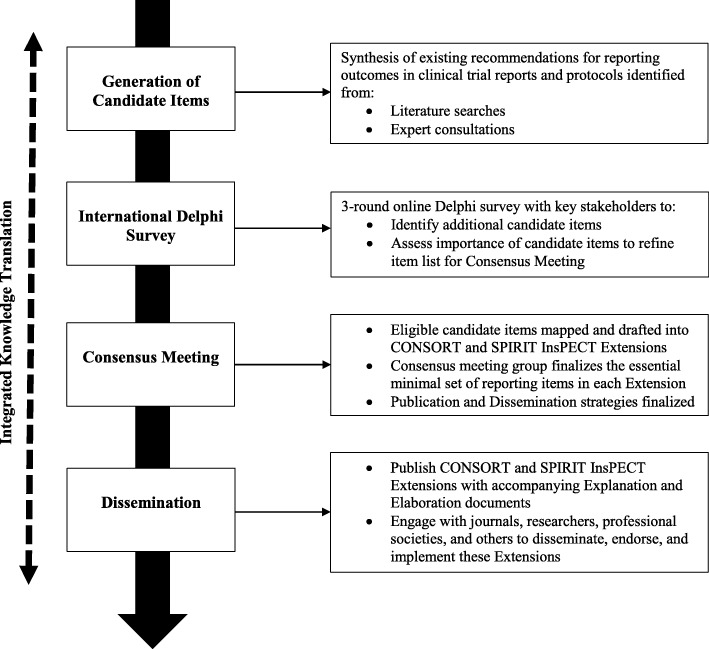
Outline of the InsPECT (Instrument for reporting Planned Endpoints in Clinical Trials) development process

The InsPECT extensions for SPIRIT and CONSORT will be developed in accordance with the reporting guideline development recommendations created by members of the Enhancing the Quality and Transparency of Health Research Quality (EQUATOR) Network [35] (Fig. 1). The development process will thus consist of three primary phases: (1) generation of candidate reporting items using a comprehensive and evidence-based approach, including literature reviews [29, 36] and expert consultations; (2) an international Delphi survey with key stakeholders to identify any additional outcome reporting items as well as to assess candidate item importance for each extension; and (3) an in-person expert consensus meeting to finalize the essential minimal set of outcome reporting items in each extension and establish post-development publication and dissemination activities.

### Initial steps and project launch

InsPECT was initially conceived as part of a Global Research in Paediatrics (GRiP) initiative in 2015 that was undertaken to develop draft recommendations for the selection and reporting of outcomes in pediatric phase II and III drug trials [29]. Through this initiative, an initial InsPECT checklist consisting of 13 candidate reporting items was developed. As reported previously [29], these items were developed based on the results of a sensitive literature search that included all existing guidelines from regulators (the US Food & Drug Administration and the European Medicines Agency) and the World Health Organization on the choice of outcomes in drug trials and a targeted Medical Literature Analysis and Retrieval System Online (MEDLINE) and Google search for existing guidelines and recommendations on outcome selection in phase II and III pediatric drug trials, as well as pilot testing with pharmaceutical industry partners and pediatric clinical trialists.

Recognizing that issues of poor outcome reporting are not unique to pediatric trials, and that there is an international need for a harmonized reporting standard for outcomes that complements existing trial reporting guidelines, the InsPECT Group assembled in 2016 to extend this initial work and develop comprehensive standards for reporting outcomes in trial protocols and reports that are generalizable and useful to trials for any population, using the EQUATOR Network reporting guideline development recommendations [35]. The InsPECT Group consists of 18 clinical trialists, clinicians, methodologists, knowledge synthesis experts, and reporting guideline developers from around the world (see Additional file 1: Table S1 for group members) with representation from key outcome methods groups (COMET, COSMIN [COnsensus-based Standards for the selection of health Measurement INstruments], and PORTal [Primary Outcomes Reporting in Trials]) and reporting standard groups (CONSORT, SPIRIT, PRISMA [Preferred Reporting Items for Systematic reviews and Meta-Analyses], and TIDieR [Template for Intervention Description and Replication]). InsPECT was registered on the EQUATOR Network on 18 November 2015 and officially launched in April 2017 after funding for InsPECT development was secured by the InsPECT Group from the Canadian Institutes of Health Research (CIHR).

### Generation of candidate items

A comprehensive approach will be used to generate candidate InsPECT items relevant to reporting outcomes in clinical trial protocols and reports, through synthesizing guidance and recommendations identified from literature reviews [29, 36] and expert consultations with the InsPECT Group. This will be led by the InsPECT Operations team, composed of the InsPECT co-chairs and project staff (see Additional file 1: Table S1). To foster collaboration and knowledge translation, iterative versions of the checklist items can be found on the Open Science Framework at https://osf.io/zgpcy/. This will be updated throughout InsPECT development.

To date, 70 preliminary candidate items have been identified through consultations with members of the InsPECT Group and a targeted examination of guidance documents published after the GRiP version of the InsPECT checklist was generated [29] (e.g., the SPIRIT-PRO and CONSORT-PRO reporting guidelines [30, 31]). With respect to InsPECT Group consultations, checklist drafts were iteratively presented and modified (i.e., addition of new candidate items and modification of existing candidate items) following feedback from members of the InsPECT Group obtained using in-person and videoconference meetings and via Research Electronic Data Capture (REDCap [37]) data management software and/or email communications. As part of an applied expert consultation, an early version of the candidate items was applied by members of the InsPECT Operations team (AC, AM) to  a clinical trial protocol and a report authored by members of the InsPECT Group (AP, SM, respectively) [38, 39]. The observed reporting results were discussed with the article authors in person, and the list of items was modified and expanded as appropriate.

As the next step in generating the InsPECT candidate item list, a comprehensive scoping review will be performed to identify and synthesize all available existing guidance on outcome reporting in clinical trials and protocols. Reporting recommendations extracted from identified guidance documents will be compared with the 70 existing candidate InsPECT items to support, refute, and refine the preliminary InsPECT items with respect to trial report and protocol reporting, and to identify additional candidate InsPECT items. The protocol for this scoping review is available elsewhere [36]. In brief, documents that provide “explicit” guidance on trial outcome reporting (“stated clearly and in detail, leaving no room for confusion or doubt” such that the guidance must specifically state that the information should be included in a clinical trial protocol or report [40]) will be searched for using (1) an electronic bibliographic database search in MEDLINE and the Cochrane Methodology Register, (2) a gray literature search, (3) documents gathered from the personal collections of expert colleagues, and (4) reference list screening. The results of this scoping review will be presented during the InsPECT Delphi process and consensus meeting and will be published in a peer-reviewed journal.

### Delphi study

We will use a three-round electronic Delphi study using a web-based questionnaire developed using REDCap [37] with a sample of expert stakeholders to identify additional candidate items and assess the importance of each candidate item for inclusion in both clinical trial protocols and clinical trial reports (Fig. 1). The Delphi study will refine the InsPECT candidate item list for evaluation at the consensus meeting. The Delphi method is an iterative multistage process that allows for consensus to be reached from a selection of disparate opinions, through structured rounds of surveys coupled with controlled feedback while maintaining anonymity [41–45].

#### Target study population and recruitment

We will engage international participants involved in the design, conduct and oversight, publication, and application of clinical trial reports and protocols to complete the Delphi study. We will specifically target the recruitment of trial report and protocol authors, reporting guideline developers, biostatisticians and epidemiologists, systematic review/meta-analysis authors, clinicians, journal editors, and research ethics board members. Participants will be purposefully sampled using a combined snowball sampling and criterion inclusion approach [46]. Relevant groups, networks, organizations, and individuals will be identified by the InsPECT Group through their professional contacts, networks, and affiliations. Published participant lists from other relevant reporting guidelines as well as author lists of relevant guidance documents identified from the scoping review will also be examined to identify potential Delphi participants. The invitation to register to participate sent to the initial list of invitees will include text that asks the recipient to share the invitation with additional qualified colleagues or relevant groups, networks, or organizations that may be interested in participating in the InsPECT Delphi study.

The inclusion criteria for participation in the Delphi study are as follows: (1) completion of a brief web-based Delphi registration survey, including agreement to complete all three rounds of the Delphi study, and (2) self-reported experience as indicated in the registration survey in any of the following activities: authoring or reviewing clinical trial protocol or reports; conducting systematic reviews/evidence synthesis of clinical trials; the design, conduct, and/or statistical planning of clinical trials; the development of a reporting guideline relevant to trial reporting; the development of a core outcome set; and/or consultation of clinical trial literature to inform clinical decision-making practices. Any registrants who indicate that they have no experience in any of these activities will be thanked for their interest in participating but will not be invited to complete the Delphi survey. There will be no geographical restrictions on eligibility. Participation in each round of the Delphi will be contingent on full completion of the prior Delphi round. Those who complete the Delphi study will receive an acknowledgement in published works for their contributions, with their permission.

Registration to complete the Delphi study will be open for approximately 1 month prior to Delphi Round 1. Registrants will be asked to provide basic demographic information, such as their job title, level of education, whether they work in an industry or academic setting, which participant group(s) they belong to (acknowledging that many individuals may represent multiple participant groups, e.g., both a trial author and a journal editor), and their relevant experience (e.g., number of trial reports written, number of trial reports reviewed). There are no guidelines for determining the number of participants in a Delphi study [45, 47]; we aim to include a sample of experts who represent diverse disciplines, organizations, and opinions. We will seek to recruit at least three to five people within each participant group; individuals who represent multiple stakeholder categories will be placed in the group with fewer participants when evaluating stakeholder representation in the completed Delphi. This redistribution will take place at the end of recruitment, according to patterns observed during the registration period. The registration list for the Delphi study will be reviewed on an ongoing basis, and recruitment strategies will be adjusted as necessary to help ensure that the relative distribution of expertise is appropriate prior to the launch of the Delphi study.

Once the Delphi study begins, eligible participants will be sent information that outlines InsPECT and the Delphi process. All instructions and survey content will be provided in English. Each round of the Delphi will stay open for approximately three weeks. Delphi participants will be instructed that the InsPECT extensions must each represent a minimal set of reporting items that are essential for reporting outcomes in trial protocols and reports, respectively. Reminder emails will be sent approximately one week before each survey closes. All participants will be allocated a unique ID number to allow identification of individual responses during survey rounds. The responses of each participant will remain anonymous throughout each survey round and will be analyzed anonymously. Only delegated members of the InsPECT Operations team will know the identifiable responses from each participant. Delphi participants will not know the identities of the other Delphi participants during the Delphi study. Participation in each survey will be voluntary. Retention between Delphi surveys will be encouraged by using recommended text [48] to convey the importance of completing the entire Delphi study.

#### Delphi procedure

Each Delphi survey will require participants to evaluate each candidate InsPECT item separately for importance in inclusion in clinical trial protocols and clinical trial reports, respectively, using a 9-point Likert scale with 1 to 3 signifying “limited importance”, 4 to 6 signifying “important but not critical”, and 7 to 9 signifying “critical” [47, 49–51]. An additional “not my expertise” option will be included to accommodate stakeholder groups that do not have the level of expertise to score all items (e.g., candidate statistical reporting items). Each item will be classified and presented as a new item (i.e., item is not part of the SPIRIT 2013 Statement or the CONSORT 2010 Statement), a revision item (i.e., concept covered in part by SPIRIT 2013 and/or CONSORT 2010), or an existing item (i.e., item or concept already part of SPIRIT 2013 and/or CONSORT 2010) to provide an opportunity for Delphi participants to confirm their inclusion and provide comments on existing items.

Consensus will be assessed using the following criteria [47, 52], consistent with methods and consensus criteria used in the development of other recent reporting guidelines [30, 53]:

1. Consensus in: ≥ 70% of participants scored the item as “critical” (score 7 to 9) and < 15% scored the item as of “limited importance” (score 1 to 3)
2. Consensus out: ≥ 70% of participants scored the item as of “limited importance” (score 1 to 3) and < 15% scored the item as “critical” (score 7 to 9)
3. No consensus: All other results.

#### Delphi Round 1

Participants will be asked to score each candidate item with respect to importance for inclusion in clinical trial protocols and clinical trial reports, respectively, to obtain a baseline measure of item importance. Free-text boxes will provide space for participants to suggest additional items and to provide comments on candidate items, including explanations to support their ratings. New item suggestions will be reviewed and integrated into Round 2 by the InsPECT Operations team. Items currently included in SPIRIT and/or CONSORT (“existing items”) that reach criteria for “consensus in” during Round 1 will be considered confirmed for inclusion in the final extensions and will not undergo additional voting or discussion. All other items will be carried forward to Round 2.

#### Delphi Round 2

Participants who completed Round 1 will be invited to complete Round 2. Participants will be provided with their Round 1 baseline score for each item and then asked to consider the aggregate group results for each item from Round 1 (e.g., median and percentage scoring each of 1–9) as well as a summary of available results of the scoping review (i.e., a summary of the empirical evidence identified to support each candidate item, if any, including any available evidence to support or refute new items suggested in Round 1, as possible) when re-scoring each item. The list of any existing SPIRIT and/or CONSORT items already meeting criteria for “consensus in” will also be provided for reference. A summary of consolidated anonymized feedback from free-text commentary will also be compiled from Round 1 and provided during Round 2. Free-text boxes will provide space for participants to provide comments on the candidate items. No additional items will be requested.

Items that meet criteria for “consensus out” in the Round 2 results will be removed from the list of candidate items. Items that meet criteria for “consensus in” or "no consensus" will move forward for consideration during the consensus meeting. Any new items suggested in Round 1 will move to Round 3 so that all items are evaluated twice. If consensus is obtained for all items during Round 2, and no new items are added in Round 1, then the Delphi study will terminate. If this occurs, the Round 2 responses will be compiled into a summary report with the same metrics calculated for Round 1 and disseminated to participants.

#### Delphi Round 3

Participants who completed Round 2 will be invited to complete Round 3. Participants will be shown the aggregate group results for each item from Round 2 and again the summary of available results of the scoping review, and their own score from Round 2. The list of any items already meeting criteria for any consensus status will also be provided. A summary of consolidated anonymized feedback from free-text commentary will also be compiled and provided from Round 2. Participants will be instructed to consider the provided information and to re-score each item. Free-text boxes will provide space for participants to provide comments on the candidate items. No additional items will be requested.

After completion of Round 3, the same analysis procedures as described in Round 2 will be employed. Items that meet criteria for “consensus out” from the Round 3 results will be removed from the list of candidate items. Items that meet criteria for “consensus in” as well as any items that did not achieve consensus will move forward for consideration during the consensus meeting.

In preparation for the consensus meeting, the InsPECT Operations team will map the remaining candidate items from the Delphi results into draft SPIRIT and CONSORT InsPECT extensions. Candidate items will be merged, and/or their phrasing clarified at this time, considering the stakeholder comments identified during the Delphi surveys. Invited consensus meeting participants will receive pre-meeting materials in preparation for the consensus meeting, which will include the drafted SPIRIT and CONSORT InsPECT candidate items, accompanied by the aggregated Delphi results and evidence summaries.

### Consensus meeting

A two day in-person consensus meeting will be held in 2019 in Toronto, Canada. Following recommended procedures for reporting guideline development [35], the primary goal will be to obtain expert group consensus on which items will be included with their finalized wording in the final InsPECT extensions for SPIRIT and CONSORT, respectively, through examination and discussion of the refined candidate item list resulting from the Delphi. This will be guided by the empirical evidence identified from the scoping review and through the opinions collected pre-meeting from the Delphi survey. The secondary goal will be to discuss and establish publication and dissemination strategies.

#### Participants

All members of the InsPECT Group will be invited to participate. We will aim to achieve a sample of 15 to 20 participants. Additional colleagues and/or Delphi participants with appropriate expertise may also be purposefully sampled and invited, if necessary to achieve adequate representation of the stakeholder groups at the consensus meeting.

#### Procedure

The consensus meeting will be led by the InsPECT chairs and an experienced independent moderator. Teleconferencing will be available for those unable to attend in person. The meeting will begin by presenting an overview of the InsPECT development process, the meeting procedures, and the meeting materials. Each candidate item for the SPIRIT and CONSORT InsPECT extensions will be presented, accompanied by the Delphi results and the evidence summaries. Moderated round table discussions of each item will follow. After discussion, anonymous voting on each item will be conducted to ensure that all voices are heard equally and for transparency in the decision-making process. Voting options for each candidate item within each guideline extension such as “Include in final checklist”, “Exclude from final checklist”, “Merge with another item”, and “Unsure” will be provided.

Items will reach consensus for inclusion when ≥ 70% of participants vote “Include in final checklist” for the item. Items will not be eligible for inclusion and will be excluded from future rounds of voting if ≥ 70% of participants vote “Exclude from final checklist”. For any items that do not reach consensus after the first round of voting, another moderated round table discussion will be held, followed by a second vote. This process will continue until either all items have reached consensus, or time has run out. All round table discussions will be audio recorded. In the event that consensus was not reached on all items at the conclusion of the meeting, the final decision for inclusion of remaining items will be made by members of the InsPECT Executive and Operations team.

Publication and dissemination strategies will also be discussed at the consensus meeting. Topics may include publication strategies, maximizing journal endorsement, end user adherence, evaluation strategy, handling criticism, and social media/web-based presence [35].

### Knowledge translation and dissemination

An integrated knowledge translation (iKT) framework for InsPECT was developed in accordance with the CIHR Guide to Knowledge Translation Planning at CIHR: Integrated and End-of-Grant Approaches [54]. iKT is defined as an ongoing relationship between researchers and decision-makers for the purpose of engaging in a mutually beneficial research project [55, 56]. Our strategy involves progress updates and feedback from members of the InsPECT Group, who are collectively representative of the project stakeholder groups. This includes at least one meeting with each member regarding project scope and methods, the opportunity to provide written feedback on an initial draft version of the checklist, and an invitation to review and provide feedback on the study protocol (this document). Ongoing iKT effort with the larger scientific community includes maintaining an active presence on social media (e.g., @InsPECT2019 on Twitter), maintaining the InsPECT website [57], and presenting InsPECT methods and preliminary results at international, national, and local conferences. InsPECT project materials are publicly available on the Open Science Framework [58].

End-of-grant activities will include publication of the InsPECT extensions for the SPIRIT and CONSORT reporting guidelines and applicable explanation and elaboration (E&E) documents. The E&Es will provide the background, rationale, and justification for each reporting item, as well as examples of good reporting. We will aim to link the InsPECT extensions via key relevant websites, such as the EQUATOR Network website [9], the SPIRIT Statement website [59], the CONSORT Statement website [60], and the COMET website [34]. Journals that currently endorse CONSORT and SPIRIT will be approached for InsPECT extension endorsement. SPIRIT and CONSORT are endorsed by roughly 100 and 600 journals, respectively [12, 61]. SPIRIT is also endorsed by regulators, funders/industry, trial groups, academic institutions, contract research organizations, and patient groups [61]. End-user feedback will be sought and incorporated, including feedback from patient groups, as appropriate, post-development. We will also follow other dissemination strategies established during the consensus meeting.

## Discussion

InsPECT will provide guidance on how to completely report any type of outcome in clinical trial reports and protocols. The development and implementation of the InsPECT extensions to CONSORT and SPIRIT have the potential to help harmonize and standardize outcome reporting across published trials reports and protocols, which will help facilitate trial reproducibility, transparency, and critical appraisal. While the emphasis of InsPECT is on clinical trials, the resultant guidance is expected to be generally applicable to all evaluative study designs, including observational studies and other study designs; future studies may develop specific InsPECT extensions for other study designs. The adoption and implementation of the InsPECT extensions will facilitate systematic reviews and meta-analyses by helping to standardize outcome reporting in the primary studies and promises to help reduce, or at least better identify, selective reporting between protocols and trial reports. The InsPECT extensions will also help increase the value of core outcome sets, which are increasingly being developed and implemented [65], by helping to enhance the clear and reproducible reporting of the core outcomes across trials and trial documents.

We expect that our implementation of a consensus-based and iKT approach will lead to improved clarity and acceptance and use of the InsPECT extensions among the broader research community. One potential challenge in the development of InsPECT is maintaining an optimal balance between usability and comprehensiveness. Different types of outcomes will require different types of information to be reported to enable reproducibility and transparency, and may also vary depending on the trial context including the intervention and population. For example, reporting on outcome assessor training may be quite relevant for some clinician-reported outcomes, but less so for biological markers such as cholesterol levels measured using standard laboratory processes. InsPECT will identify the minimal level of information required to be reported. Involving a large, diverse, and international group of stakeholders in the development of InsPECT may increase usability among the broader research community. As of September 2018, an initial list of InsPECT candidate items has been generated and the scoping review is ongoing. The Delphi study will be completed prior to the 2019 consensus meeting.

### Trial status

Not applicable.

## Additional files

Additional file 1: **Table S1.** Group membership for the development of the Instrument for Reporting of Planned Endpoints in Clinical Trials (InsPECT). (DOCX 22 kb)

## Abbreviations

CIHR: Canadian Institutes of Health Research
COMET: Core Outcome Measures in Effectiveness Trials
CONSORT: Consolidated Standards of Reporting Trials
E&E: Explanation and elaboration
EQUATOR: Enhancing the Quality and Transparency of Health Research
iKT: Integrated knowledge translation
InsPECT: Instrument for reporting Planned Endpoints in Clinical Trials
SPIRIT: Standard Protocol Items: Recommendations for Interventional Trials
