# Supplementary material for: Improving outcome reporting in clinical trial reports and protocols: study protocol for the Instrument for reporting Planned Endpoints in Clinical Trials (InsPECT)
Source: Trials. 2019 Mar 6;20:161. doi: 10.1186/s13063-019-3248-0 (PMC6404348; doi:10.1186/s13063-019-3248-0)
Supplement: Supplementary file 1 — Table S1. Group membership for the development of the Instrument for Reporting of Planned Endpoints in Clinical Trials (InsPECT). (DOCX 22 kb) [file 13063_2019_3248_MOESM1_ESM.docx]

**Additional file 1**

**Table S1** Group membership for the development of the Instrument for Reporting Planned Endpoints in Clinical Trials (InsPECT).

| **Name** | **InsPECT Role**^a^ | **Primary Affiliations** |
| --- | --- | --- |
|  |  |  |
| Martin Offringa | Co-chair | The Hospital for Sick Children, Toronto, Canada |
| Nancy Butcher | Co-chair | The Hospital for Sick Children Research Institute, Toronto, Canada |
| David Moher | InsPECT Executive | Centre for Journalology, Clinical Epidemiology Program, Ottawa Hospital Research Institute, Ottawa, Canada |
| An-Wen Chan | InsPECT Executive | Women’s College Research Institute, Toronto, Canada |
| Peter Szatmari | InsPECT Executive | Centre for Addiction and Mental Health, Toronto, Canada |
| Agostino Pierro | InsPECT Executive | The Hospital for Sick Children, Toronto, Canada |
| Lauren Kelly | InsPECT Executive | The Children’s Hospital Research Institute of Manitoba, Manitoba, Canada |
| Mufiza Kapadia | InsPECT Executive | The Hospital for Sick Children Research Institute, Canada  Roche Canada, Canada (current) |
| Lisa Askie | InsPECT Group | National Health and Medical Research Centre Clinical Trials Centre, Australia |
| P.J. Devereaux | InsPECT Group | Population Health Research Institute, Hamilton, Canada |
| Dean Fergusson | InsPECT Group | Ottawa Hospital Research Institute, Ottawa, Canada |
| Paul Glasziou | InsPECT Group | Bond University, Australia |
| Jeremy Grimshaw | InsPECT Group | Ottawa Hospital Research Institute, Ottawa, Canada |
| Suneeta Monga | InsPECT Group | The Hospital for Sick Children, Toronto, Canada |
| Caroline Terwee | InsPECT Group | Amsterdam UMC, location VUmc, Department of Epidemiology and Biostatistics, The Netherlands |
| Wendy Ungar | InsPECT Group | The Hospital for Sick Children, Toronto, Canada |
| Sunita Vohra | InsPECT Group | University of Alberta, Edmonton, Canada |
| Paula Williamson | InsPECT Group | University of Liverpool, UK |
| Emma Mew | Operations Team | Clinical Research Project Manager, The Hospital for Sick Children Research Institute, Toronto, Canada |
| Andrea Monsour | Operations Team | Clinical Research Project Coordinator, The Hospital for Sick Children Research Institute, Toronto, Canada |
| Alyssandra Chee-A-Tow | Operations Team | Clinical Research Project Coordinator, The Hospital for Sick Children Research Institute, Toronto, Canada |
| Leena Saeed | Operations Team | Clinical Research Project Assistant, The Hospital for Sick Children Research Institute, Toronto, Canada |

^a^Drs. Offringa and Butcher co-chair all sub-committees, including the InsPECT Executive, Operations Team, and the InsPECT Group. All members of the InsPECT Executive are also part of the InsPECT Group. For details on roles, see text.
